# EDSS Change Relates to Physical HRQoL While Relapse Occurrence Relates to Overall HRQoL in Patients with Multiple Sclerosis Receiving Subcutaneous Interferon ***β***-1a

**DOI:** 10.1155/2015/631989

**Published:** 2015-07-05

**Authors:** Barbara G. Vickrey, Liesly Lee, Fraser Moore, Patrick Moriarty

**Affiliations:** ^1^Department of Neurology, University of California, Los Angeles, 710 Westwood Plaza, Los Angeles, CA 90095-1796, USA; ^2^Greater Los Angeles VA Medical Center, 11301 Wilshire Boulevard, Los Angeles, CA 90073, USA; ^3^Division of Neurology, University of Toronto, Sunnybrook Health Science Center, 2075 Bayview Avenue, Toronto, ON, Canada M4N 3M5; ^4^Department of Neurology, Jewish General Hospital, McGill University, 3755 Côte-Sainte-Catherine Road, Montreal, QC, Canada H3T 1E2; ^5^Department of Medical Affairs, EMD Serono, 2695 North Sheridan Way, Suite 200, Mississauga, ON, Canada L5K 2N6

## Abstract

*Objective*. To compare patterns of associations of changes in mental and physical health dimensions of health-related quality of life (HRQoL) over time with relapse occurrence and changes in Expanded Disability Status Scale (EDSS) scores in patients with relapsing multiple sclerosis (RMS). *Methods*. This 24-month, phase IV, observational study enrolled 334 patients with RMS who received interferon *β*-1a 44 *μ*g or 22 *μ*g subcutaneously three times weekly. At each 6-month visit, patients completed the Multiple Sclerosis Quality of Life-54 (MSQOL-54) and site investigators assessed EDSS and recorded relapse occurrence. A generalized linear model procedure was used for multivariable analyses (per protocol) that explored unique associations of EDSS score change and relapse occurrence with MSQOL-54 physical health composite score (PCS) and mental health composite score (MCS). *Results*. HRQoL improved over 2 years among those who completed the study. Occurrence of ≥1 relapse was significantly associated with lower MSQOL-54 PCS and MCS. Changes in EDSS score were significantly associated with MSQOL-54 PCS, but not MCS. *Conclusions*. HRQoL assessments, particularly those that examine mental health, may provide information on the general health status of patients with RMS that would not be recognized using traditional clinician-assessed measures of disease severity and activity. This trial is registered with ClinicalTrials.gov; identifier: NCT01141751.

## 1. Introduction

Clinical assessment of patients with multiple sclerosis (MS) typically focuses on observer-based measures of physical disability. However, there is an acknowledged need to also monitor patient-reported health-related quality of life (HRQoL) over time when evaluating disease progression and making treatment and management decisions [1]. Patient-reported HRQoL provides a broad measure of disease impact (including health dimensions that cannot be evaluated using observer-based measures of physical disability) [2], may predict future disability progression [3, 4], and is associated with lesion burden and brain atrophy measured using magnetic resonance imaging [5]. The COMPARE study was primarily designed to assess and compare the usefulness and psychometric properties of two HRQoL instruments in a large cohort of patients with relapsing MS treated with subcutaneous interferon *β*-1a; primary and secondary endpoint results from this study have been reported previously [6]. The COMPARE study also offered the opportunity for analyses of associations of changes over time in different dimensions of HRQoL with changes in traditional clinician-assessed parameters of disease severity and activity. Here, we report the results of these analyses.

## 2. Methods

### 2.1. Study Design

The design and conduct of the COMPARE study have been described previously [6]. Briefly, COMPARE was a 24-month, phase IV, observational, open-label, single-arm study conducted across 34 MS clinics in Canada (ClinicalTrials.gov identifier: NCT01141751). The study enrolled patients who had a confirmed diagnosis of relapsing MS according to the McDonald (2001) diagnostic criteria [7] and were eligible for, and willing to start, treatment with interferon *β*-1a subcutaneously three times weekly as prescribed by their treating physician. Patients were excluded if they had taken a disease-modifying drug (DMD) within the last month (or 30 days) before study entry. All patients received treatment with interferon *β*-1a 44 *μ*g or 22 *μ*g subcutaneously three times weekly. Clinic visits were scheduled at baseline and at 6, 12, 18, and 24 months. Patients who withdrew from the study were invited to return for an early termination visit. At each visit, patients completed the Multiple Sclerosis Quality of Life-54 (MSQOL-54) questionnaire, an HRQoL measure that yields summary scores for physical health (physical health composite score [PCS]) and mental health (mental health composite score [MCS]) [8]. Both PCS and MCS are expressed on a scale of 0 (poorest quality of life) to 100 (best possible quality of life). Site investigators also rated patients' Expanded Disability Status Scale (EDSS) scores at each visit. The occurrence of relapses since the previous visit was recorded at all postbaseline visits. The study was conducted in accordance with the Declaration of Helsinki (2004), International Conference on Harmonization Harmonized Tripartite Guideline for Good Clinical Practice, and local regulations. An institutional review board or independent ethics committee approved the protocol at each center before study initiation. All patients gave written informed consent.

### 2.2. Statistical Analyses

The baseline characteristics of patients with and without Month 24 or early termination visits were compared using the two-sided Mann-Whitney *U* test (quantitative variables) or two-sided Fisher's exact test (qualitative variables). Paired changes from baseline to 6, 12, 18, and 24 months in MSQOL-54 PCS and MCS and EDSS scores were analyzed using two-sided Wilcoxon matched pairs signed-rank test. The generalized linear model procedure was used for multivariable analyses. In the models used to explore unique associations of EDSS progression with HRQoL, MSQOL-54 PCS or MCS at 6, 12, 18, or 24 months was included as a dependent variable, and ≥1- or ≥1.5-point increase in EDSS score from baseline to the respective follow-up visit was included as an independent variable. In the models used to explore unique associations of relapse occurrence with HRQoL, MSQOL-54 PCS or MCS at 12 or 24 months was included as a dependent variable, and occurrence of one or more relapses from baseline to 12 months or after Month 12 to 24 months, respectively, was included as an independent variable. All models included age, sex, highest level of education (categorized as either not greater than or greater than secondary), whether DMD-naïve at study onset, whether terminated the study early, and baseline HRQoL measure score as independent variables, except for the analysis with relapses after Month 12 to 24 months, in which the HRQoL measure score at 12 months was an independent variable.

## 3. Results

### 3.1. Patients

Of 334 patients enrolled in the study, 196 (59%) completed the end-of-study visit at 24 months [6]. Of the 138 patients who withdrew from the study before 24 months, 81 (59%) attended an early termination visit. The mean (standard deviation [SD]) time on study was 19.5 (7.2) months (median [range] 23.5 [0.0–30.8] months). Baseline demographic and disease characteristics of the overall study cohort, together with characteristics for subgroups of patients with and without data from Month 24 or early termination visits, are presented in Table 1. Baseline HRQoL scores are presented in Table 2. The majority of patients (80%) were DMD-naïve and 75% had a baseline EDSS score ≤2.5 (Table 1).

### 3.2. HRQoL, EDSS, and Relapses over Time

Significant mean paired improvements were observed both in MSQOL-54 PCS and in MSQOL-54 MCS from baseline to 12 months and from baseline to 24 months (*p* < 0.03 for PCS; *p* ≤ 0.0003 for MCS; Table 2). A small but significant improvement in EDSS score was seen from baseline to 12 months (*p* < 0.05), while mean paired changes in EDSS score from baseline to 24 months and from baseline to the early termination visit were not significant (Table 2). At least one relapse was reported in 86 (26%) of 334 patients between baseline and 12 months and in 50 (21%) of 241 patients after Month 12 to 24 months.

### 3.3. Unique Associations of EDSS Progression and Relapse Occurrence with HRQoL

An increase in EDSS score of ≥1 point (Table 3) or ≥1.5 points (data not shown) was significantly associated with lower (worse) MSQOL-54 PCS at all time points (*p* < 0.04 for all), except at 12 months for an increase in EDSS score of ≥1.5 points. There was a significant association between lower MCS and an increase in EDSS score of ≥1.5 points at 24 months (*p* < 0.01); there were no significant associations between MCS and an increase in EDSS score of ≥1.5 points at other time points or for an increase in EDSS score of ≥1 point (Table 3). The occurrence of one or more relapses between baseline and 12 months was significantly associated with both lower (worse) follow-up PCS (*p* = 0.005) and MCS (*p* < 0.05; Table 4). There were no significant associations between the occurrence of one or more relapses after Month 12 to 24 months and follow-up PCS or MCS (Table 4).

## 4. Discussion

In this observational study of patients with relapsing MS treated with interferon *β*-1a 44 *μ*g or 22 *μ*g subcutaneously three times weekly, patient-reported HRQoL appeared to improve over 2 years for those completing the study. This observation is consistent with previous studies suggesting that subcutaneous interferon *β*-1a therapy may have a positive effect on the HRQoL of patients with relapsing-remitting MS [9, 10], although attribution is not possible in the absence of a concurrent nontreatment comparator group.

Results from our prespecified analyses showed that changes in EDSS score were associated with changes in the physical but not the mental health dimension of HRQoL; therefore, the physical components of the HRQoL score over time appear to correspond with the clinical situation of the patient in terms of physical disability as evaluated by an external rater. In contrast, the occurrence of one or more relapses during the first 12 months was associated with poorer outcomes not only in physical health but also in mental health dimensions of HRQoL. The lack of association between EDSS change and mental health aspects of HRQoL at most time points is likely a reflection of MS-related impairments, such as fatigue and depression, which are not or are only minimally evaluated by EDSS [11].

Regarding limitations, we note that we did not capture data on whether study participants had a clinical diagnosis of depression, which is an important contributor to HRQoL in MS. However, the MSQOL-54 mental health composite score does include items on depressive symptomatology and thus taps into the construct of depression. To minimize missing data, we invited study participants who left the study early to attend an early termination visit. Fifty-nine percent of those patients who withdrew from the study before 24 months attended the early termination visit; therefore, we were able to include early termination visit data in our analyses, covarying for early termination status in multivariable models. Neither Month 24 nor early termination visit data were available for 17% (57/334) of patients; however, the baseline characteristics of patients with and without Month 24 or early termination visits were not significantly different (Table 1).

Another limitation of the present study was entry criteria, which specified that while patients should be willing to start medication, they should not be receiving DMD treatment; our patient cohort did not include more severely disabled patients, and half of the patients had been diagnosed with MS within the previous 4 months. Consequently, the findings of the exploratory analyses may not be applicable to patients with relapsing MS who have a greater level of disability or patients at a later stage of disease. Finally, we acknowledge that the interpretation of changes in HRQoL over time can also be complicated by “response shift,” whereby HRQoL self-reported by the patient might be influenced by psychological phenomena such as adaptation to the disease [1].

## 5. Conclusions

Prespecified multivariable analyses from a 24-month observational study in patients with relapsing MS receiving subcutaneous interferon *β*-1a suggest that, over the initial 12 months following enrollment, EDSS changes are solely associated with the physical health domain of HRQoL, but relapse occurrence is broadly associated with both physical and mental health aspects of HRQoL.

## Figures and Tables

**Table 1:** Baseline demographics and disease characteristics [6].

Baseline characteristic	Patients with data at Month 24 or ET visit (*n* = 277)	Patients without data at Month 24 or ET visit (*n* = 57)	Overall (*N* = 334)
Age, years, mean (SD)	39.0 (9.3)	37.1 (9.1)	38.7 (9.3)
Female, *n* (%)	212 (76.5)	42 (73.7)	254 (76.0)
Caucasian, *n* (%)	257 (92.8)	52 (91.2)	309 (92.5)
Employment status, *n* (%)			
Student	16 (5.8)	4 (7.0)	20 (6.0)
Full-time, part-time, or retired workers	194 (70.0)	33 (57.9)	227 (68.0)
Unemployed	66 (23.8)	20 (35.1)	86 (25.7)
Homemaker	1 (0.4)	0 (0.0)	1 (0.3)
Highest level of education achieved, *n* (%)			
Primary or secondary	119 (43.0)	25 (43.9)	144 (43.1)
Tertiary or above	158 (57.0)	32 (56.1)	190 (56.9)
MS classification, *n* (%)			
Relapsing-remitting	265 (95.7)	54 (94.7)	319 (95.5)
Secondary progressive	4 (1.4)	2 (3.5)	6 (1.8)
Clinically isolated syndrome	8 (2.9)	1 (1.8)	9 (2.7)
Time since onset of MS symptoms, months, median (range)	25.0 (0–422)	31.0 (4–251)	26.0 (0–422)
Time since MS diagnosis, months, median (range)	4.0 (0–386)	5.0 (0–203)	4.0 (0–386)
Previously received DMDs, *n* (%)	53 (19.1)	15 (26.3)	68 (20.4)
EDSS score, *n* (%)^a^			
0	32 (11.6)	7 (12.3)	39 (11.7)
1.0–1.5	102 (36.8)	14 (24.6)	116 (34.7)
2.0–2.5	83 (30.0)	14 (24.6)	97 (29.0)
3.0–3.5	33 (11.9)	13 (22.8)	46 (13.8)
4.0–4.5	14 (5.1)	3 (5.3)	17 (5.1)
5.0–5.5	7 (2.5)	2 (3.5)	9 (2.7)
6.0–6.5	6 (2.2)	4 (7.0)	10 (3.0)
≥7.0	0	0	0
Mean (SD)	2.0 (1.3)	2.4 (1.6)	2.0 (1.4)

There were no significant differences in the baseline characteristics of patients with and without Month 24 or ET visits; baseline characteristics were compared using the 2-sided Mann-Whitney *U* test (quantitative variables) or 2-sided Fisher's exact test (qualitative variables).

^a^Percentages add up to values slightly above 100% (100.1%) due to rounding up to one decimal place.

DMD, disease-modifying drug; EDSS, Expanded Disability Status Scale; ET, early termination; MS, multiple sclerosis; SD, standard deviation.

**Table 2:** Mean values and paired changes from baseline in MSQOL-54 PCS and MCS and in EDSS scores.

Dimension	Baseline	12 months	24 months	Early termination
MSQOL-54 PCS				
*n*	313	238	182^a^	68
Mean (SD)	60.9 (19.7)	65.0 (20.0)	64.9 (20.3)	60.7 (22.8)
Paired change from baseline				
*n*	—	230	174^a^	66
Mean (SD)	—	2.0 (14.3)	2.2 (15.3)	0.3 (14.4)
*p* value^b^	—	0.014	0.027	NS

MSQOL-54 MCS				
*n*	331	249	191^a^	73
Mean (SD)	65.6 (20.5)	71.4 (19.7)	71.7 (20.0)	67.3 (22.5)
Paired change from baseline				
*n*	—	248	190^a^	72
Mean (SD)	—	3.7 (17.5)	4.3 (19.1)	1.3 (19.2)
*p* value^b^	—	<0.0001	0.0003	NS

EDSS score				
*n*	334	251	196	73
Mean (SD)	2.0 (1.4)	1.8 (1.3)	1.9 (1.4)	2.2 (1.8)
Paired change from baseline				
*n*	—	251	196	73
Mean (SD)	—	−0.1 (0.9)	0.1 (1.0)	0.1 (1.0)
*p* value^b^	—	0.047	NS	NS

MCS and PCS are expressed on a scale of 0 (poorest quality of life) to 100 (best possible quality of life). Paired changes from baseline to 24 months and ET in MSQOL-54 PCS and MCS and EDSS scores were also previously reported in Moore et al. [6].

^a^ *n* numbers lower than 196 (number of patients who completed the 24-month study); data for these endpoints were not available for all patients who completed the study.

^b^ *p* value calculated using a two-sided Wilcoxon matched pairs signed-rank test.

EDSS, Expanded Disability Status Scale; ET, early termination; MCS, mental health composite score; MSQOL-54, Multiple Sclerosis Quality of Life-54; NS, not statistically significant; PCS, physical health composite score; SD, standard deviation.

**Table 3:** Results of multivariable analyses: unique associations of EDSS score increase of ≥1 point with follow-up MSQOL-54 PCS and MCS.

	Estimate (SE)	*t*-value	*p* value
6 months			
MSQOL-54 PCS, *n* = 279			
New to DMD	−4.53 (1.89)	−2.40	0.017
EDSS change ≥1 point	−**7.95 (2.34)**	−**3.40**	**0.0008**
MSQOL-54 MCS, *n* = 302			
Greater than secondary education	5.00 (1.80)	2.78	0.006
EDSS change ≥1 point	−4.40 (2.82)	−1.56	NS

12 months			
MSQOL-54 PCS, *n* = 248			
EDSS change ≥1 point	−**5.34 (2.49)**	−**2.14**	**0.033**
MSQOL-54 MCS, *n* = 269			
Greater than secondary education	4.07 (1.99)	2.05	0.042
EDSS change ≥1 point	−2.88 (2.75)	−1.05	NS

18 months			
MSQOL-54 PCS, *n* = 210			
Age	−0.34 (0.12)	−2.86	0.005
EDSS change ≥1 point	−**7.36 (2.84)**	−**2.59**	**0.010**
MSQOL-54 MCS, *n* = 229			
New to DMD	6.80 (2.89)	2.35	0.019
EDSS change ≥1 point	−4.35 (3.04)	−1.43	NS

24 months			
MSQOL-54 PCS, *n* = 181			
EDSS change ≥1 point	−**6.70 (2.74)**	−**2.45**	**0.016**
MSQOL-54 MCS, *n* = 196			
Greater than secondary education	6.03 (2.53)	2.38	0.018
EDSS change ≥1 point	−2.67 (2.97)	−0.90	NS

Significant associations between EDSS change ≥1 point and follow-up MSQOL-54 PCS or MCS are highlighted in bold. The generalized linear model procedure was used. All models included age, sex, highest level of education (not greater than or greater than secondary), whether naïve to DMD at study onset, whether terminated the study early, and baseline HRQoL measure score. Baseline HRQoL measure score was significant in all models.

DMD, disease-modifying drug; EDSS, Expanded Disability Status Scale; HRQoL, health-related quality of life; MCS, mental health composite score; MSQOL-54, Multiple Sclerosis Quality of Life-54; NS, not statistically significant; PCS, physical health composite score; SE, standard error.

**Table 4:** Results of multivariable analyses: unique associations of relapse occurrence with follow-up MSQOL-54 PCS and MCS.

	Estimate (SE)	*t*-value	*p* value
One or more relapses from baseline to 12 months			
MSQOL-54 PCS, *n* = 242			
≥1 relapse	−**5.41 (1.92)**	−**2.82**	**0.005**
MSQOL-54 MCS, *n* = 264			
Greater than secondary education	4.15 (2.00)	2.08	0.039
≥1 relapse	−**4.33 (2.17)**	−**2.00**	**0.047**

One or more relapses after Month 12 to 24 months^a^			
MSQOL-54 PCS, *n* = 117			
Age	−0.28 (0.10)	−2.76	0.006
≥1 relapse	−2.98 (2.31)	−1.29	NS
MSQOL-54 MCS, *n* = 191			
Age	−0.25 (0.11)	−2.28	0.024
Greater than secondary education	4.42 (2.15)	2.05	0.042
≥1 relapse	−3.39 (2.50)	−1.36	NS

Significant associations between relapse occurrence and follow-up MSQOL-54 PCS or MCS are highlighted in bold. The generalized linear model procedure was used; only associations at *p* ≤ 0.05 are listed except for relapse occurrence, which is listed in the table regardless of significance. All models included age, sex, highest level of education (not greater than or greater than secondary), whether naïve to DMD at study onset, whether terminated the study early, and baseline HRQoL measure score. Baseline HRQoL measure score was significant in all models.

^a^The MSQOL-54 PCS or MCS at 12 months was considered the baseline HRQoL measure score.

DMD, disease-modifying drug; HRQoL, health-related quality of life; MCS, mental health composite score; MSQOL-54, Multiple Sclerosis Quality of Life-54; NS, not statistically significant; PCS, physical health composite score; SE, standard error.
